# A critical evaluation of the effectiveness of interventions for improving the well-being of caregivers of children with cerebral palsy: a systematic review protocol

**DOI:** 10.1186/s13643-016-0287-4

**Published:** 2016-07-13

**Authors:** Jermaine M. Dambi, Jennifer Jelsma, Tecla Mlambo, Matthew Chiwaridzo, Cathrine Tadyanemhandu, Mildred T. Chikwanha, Lieselotte Corten

**Affiliations:** Department of Health and Rehabilitation Sciences, Faculty of Health Sciences, University of Cape Town, Anzio Road, Observatory, Cape Town, South Africa; Department of Rehabilitation, College of Health Sciences, University of Zimbabwe, P.O. Box AV178, Avondale, Harare Zimbabwe; University of Witwatersrand, Johannesburg, South Africa; Stellenbosch University, Stellenbosch, South Africa

**Keywords:** Cerebral palsy, Caregiver, Program, Intervention, Burden/strain, Quality of life, Well-being, Evaluation, Systematic review, Protocol

## Abstract

**Background:**

Over the years, family-centered care has evolved as the “gold standard” model for the provision of healthcare services. With the advent of family-centered approach to care comes the inherent need to provide support services to caregivers in addition to meeting the functional needs of children with physical disabilities such as cerebral palsy (CP). Provision of care for a child with CP is invariably associated with poor health outcomes in caregivers. As such, there has been a surge in the development and implementation of interventions for improving the health and well-being of these caregivers. However, there is a paucity of the collective, empirical evidence of the efficacy of these interventions. Therefore, the broad objective of this review is to systematically review the literature on the effectiveness of interventions designed to improve caregivers’ well-being.

**Methods/design:**

This is a systematic review for the evaluation of the effectiveness of interventions designed to improve caregivers’ well-being. Two independent, blinded, reviewers will search articles on PubMed, Scopus, Web of Science, CINAHL, Psych Info, and Africa-Wide Information using a predefined criterion. Thereafter, three independent reviewers will screen the retrieved articles. The methodological quality of studies meeting the selection criterion will be evaluated using the Briggs Institute checklists. Afterwards, two independent researchers will then apply a preset data-extraction form to collect data. We will perform a narrative data analysis of the final sample of studies included for the review.

**Discussion:**

The proposed systematic review will provide the empirical evidence of the efficacy of interventions for improving the well-being of caregivers of children with physical disabilities. This is important given the great need for evidenced-based care and the greater need to improve the health and well-being of caregivers.

**Systematic review registration:**

PROSPERO CRD42016033975

**Electronic supplementary material:**

The online version of this article (doi:10.1186/s13643-016-0287-4) contains supplementary material, which is available to authorized users.

## Introduction

### Background

Provision of care to a child with a chronic/long-term health condition has been extensively demonstrated to negatively affect the health and health-related quality of life (HRQoL) of informal caregivers [1–3]. This is exacerbated if the child suffers from a complex neurodevelopmental condition such as cerebral palsy (CP) [2, 3]. Children with CP most often present with multiple functional problems and invariably require assistance in day-to-day activities such as feeding, bathing, among others [4]. Furthermore, children with CP require frequent medical care necessitating the caregivers to visit health institutions thus compounding the magnitude of the perceived burden of care [5]. Consequently, caregiving for a child with CP is often associated with anxiety [6–8], stress [9, 10], depression [8, 11–13], low self-efficacy [14], financial burden [15, 16], musculoskeletal pain [13, 17], poor physical health, and lower HRQoL in informal caregivers [3, 18]. It is also known that the functional limitations of the children become more apparent as the child grows older, increasing the magnitude of caregiver burden with the passage of time [1, 3, 5, 18].

Over the past few decades, there has been a paradigm shift in the provision of rehabilitation services with the family-centered approach evolving as the “gold standard” model of service delivery [19, 20]. There is now a stronger emphasis to provide support services for caregivers in addition to meeting the functional needs of children with CP [19, 21]. This is justified because there is evidence that poor caregiver health is associated with poorer functional outcomes in children with CP [3, 22]. For optimal rehabilitation outcomes to be achieved, the caregiver is expected to be compliant with attendance to appointment schedules and with instructions regarding facilitation to improve functioning by expediently implementing the prescribed home exercise program [23]. This is important as the neuroplasticity theory emphasizes the importance of constant practice of functional activities in children with CP (after suffering from brain injury), to improve function [24], hence, the need for continued care at home by the caregivers after the hospital-based therapy sessions for optimal functional outcomes. On the same wavelength, economic evaluations in the USA alone have pegged services of informal caregivers at around $450 billion in 2011 [25]. This undoubtedly demonstrates that informal caregivers are a vital human resource in the management of patients with long-term health conditions [21, 26, 27].

To this end, there have been attempts to develop supportive interventions aimed at increasing caregiver well-being for moral, ethical, and economic reasons [1, 3, 25]. The most commonly cited interventions include educational strategies, e.g., caregivers’ training workshops, and psychosocial strategies such as counselling, support groups, cognitive behavioral techniques, and respite care, among others [28–31]. Felicity et al. [28] performed a randomized controlled trial (RCT) in Australia to determine the impact of a family behavioral interventions such as the acceptance and commitment therapy on the well-being of caregivers of children with traumatic brain injury (*N* = 59). The results indicated that family behavioral interventions may lead to improvements in caregivers’ self-efficacy, confidence, family adjustment, and psychosocial well-being. Moreover, support groups have been demonstrated to lead to decreases in parental stress, increased psychosocial well-being in domains such as hope, happiness, and self-esteem in Hong Kong caregivers of children with CP [30].

In another study, 26 Indian caregivers of newly diagnosed children with CP were exposed to an educational program aimed at increasing parental knowledge of CP [29]. The results revealed that even a single session is enough to increase caregivers’ knowledge. Additionally, the authors postulated that knowledgeable caregivers were most likely to adjust to the demands of caregiving [29]. This is further substantiated by findings from a study on 53 Indian caregivers of children with CP. In the aforementioned study, caregivers’ level of knowledge of CP was improved after they were exposed to an educational film [31].

However, there is no collective empirical evidence to determine the efficacy of these interventions in improving caregivers’ well-being. Additionally, given the heterogeneity of methodologies applied, it is thus important to systematically evaluate the impact of these interventions on caregivers’ well-being. Above all, there is an urgent need for evidence-based practice in implementing supportive interventions for caregivers of children with long-term disabilities.

### Objectives

Therefore, the broad objective of this review is to systematically evaluate the effectiveness of interventions designed to improve caregivers’ well-being. The specific objectives are to:

1. Identify interventions targeted at improving caregivers’ well-being
2. Describe the identified interventions targeted at improving caregivers’ well-being
3. Identify the components of the interventions deemed to be effective in improving caregivers’ well-being

## Methods

### Study registration

The protocol was structured according to the Preferred Reporting Items of Systematic Reviews and Meta-Analyses Protocol (PRISMA-P) guidelines [32] and has been registered on PROSPERO database (Ref: CRD42016033975).

### Eligibility criteria

In selecting the studies, we will apply the following criteria:

#### Participants

A previously described selection criterion will be utilized [33]. Studies will be included if the primary respondents were informal caregivers, aged 18 years and above, providing care for children with chronic/long-term health conditions in the age range 0–12 years. The dynamics of caregiving are dependent on the developmental stage/chronological age of a child with a physical disability. For instance, physical burden and the overall burden of caregiving is likely to increase as child gets older and heavier and as they enter the teenage years [22]. Additionally, we will include studies on caregivers of children with physical disabilities such as CP, spina bifida, hydrocephalus, and other long-term health conditions such as perinatal stroke, developmental delay, and traumatic brain injury among others. We envisage that the dynamics of providing care for a child with functional limitations are the same, regardless of the causative agent [33]. There will be no limit as to the severity of disability for the studies to be selected. Additionally, for this review, an informal caregiver denotes someone not formally trained and remunerated for assuming the caregiving role. To minimize the effects of confounding variables, studies with caregivers (i) suffering from chronic conditions and (ii) providing care for other children below the age of five or chronically ill relative/spouse will be excluded.

#### Study designs

Due to a dearth of RCTs on interventions of caregivers of children with CP, which was revealed in a pilot/preliminary literature search, all quantitative study designs will be considered in this review. Similarly, the preliminary search revealed a paucity in qualitative studies thus the decision to include quantitative designs only.

#### Interventions and outcomes

All studies aimed at either development or implementation of interventions for improving the well-being of caregivers of children with a chronic/long-term health condition as exemplified in Table 1 will be included.

**Table 1:** PICO table

Population	Interventions		Comparison	Outcomes	
	Intervention category	Example(s)		Example(s) outcome(s)	Example(s) of outcome measures
• Primary caregivers • Informal/unpaid caregivers • 18 years and above	Psychosocial	Counselling	Non-exposed controls	Caregiver burden/caregiver strain	1. Caregiver strain index 2. Caregiver burden scale
	Psychosocial	Group therapy sessions	Caregivers of healthy children	HRQoL	1. EQ-5D 2. SF-36 3. WHO QOLBREF
	Psychosocial	Cognitive behavioral techniques	Caregivers of children with minor illness	Depression	Becky depression scale
	Psychosocial	Cognitive behavioral techniques		Anxiety	Hospital anxiety and depression scale (HADS)
	Psychosocial	Cognitive behavioral techniques		Self-efficacy	General self-efficacy scale
					General perceived self-efficacy scale
	Educational	Oral lectures		Knowledge/information on cerebral palsy	Knowledge of cerebral palsy questionnaire
	Physical activity	Exercise classes		Physical health levels	Borg scale

#### Setting

All study settings will be considered.

#### Language

We will only consider full articles published in English, German, French, and Dutch languages. Due to financial challenges, we do not have resources for paying for the translation of articles published in any other language than these.

## Information sources

### Search strategy

The following databases will be searched: PubMed, EMBASE, Scopus, MEDLINE, Psych INFO, CINAHL, Cochrane library, PEDro, and OT seeker. As this is a scoping review, we will not impose a time limit as to the data of publication of the articles to gather as much empirical evidence as possible. We will also perform manual searches of references of identified articles to search for additional publications. The following key terms will be utilized in search of the literature: “caregivers” OR “care*” OR “mother*” AND “cerebral palsy” OR “physical disability*” AND (“physiotherapy” OR “physical therapy” OR “rehabilitation” OR Occupational therapy) AND “intervention*” OR “treat*” OR “prog*” Outlined in Table 2 is an example of how we will search for the articles in CINAHL database.

**Table 2:** Search strategy

Key word	Alternative words
caregiver	carer* OR mother* OR parent* OR legal guardian*
children	child* OR paediatric* OR toddler* OR infant* OR pediatric*
cerebral palsy	CP OR physical disability OR disability* OR neurodev* disorder*OR traumatic brain injur*
burden	strain OR stress OR burnout
health related quality of life	quality of life OR HRQoL OR well-being OR wellness
interventions	program*
evaluation	determination OR measurement
physiotherapy	physical therapy OR rehabilitation OR occupational therapy OR physical medicine OR physiatrist

### Study records

#### Data management

Retrieved articles will be imported into RevMan (version 5.3) which is a data management program. The electronic searches will also be saved on users’ PubMed, Scopus, and EBSCOhost accounts. The principal researcher will create a shared Dropbox folder to facilitate collaboration among reviewers during the data collection and extraction process. Summaries of all the searches will be printed and are to be used as physical backup for the screened articles.

#### Selection and data collection process

The principal author (JD) and a second reviewer (MC) will independently search the databases and extract the titles and abstracts for further investigation using a predefined search strategy. Thereafter, two researchers (MTC and CT) will independently apply the predefined selection criterion and retrieve the full manuscripts of articles deemed relevant. Disagreements during the article screening stage will be settled through discussions, and where appropriate, an independent researcher (JJ) not involved in the searching and screening of the articles will make the final decision. The principal researcher (JD) will manually search the reference lists of identified articles to screen for potential articles for inclusion in the systematic review. Lastly, two independent reviewers (LC and TM) will blindly screen the retrieved articles using a standardized data collection form. The two data collection sheets will be reconciled into one data set through discussions between the principal author (JD) and two reviewers (LC and TM). One independent reviewer (JJ) will make the final decision if there be any disagreements. Information to be extracted will include the research setting and design, study sample, demographics of the participants, outcome measures, types of interventions, and results.

### Outcomes and prioritization

#### Primary outcomes

For this review, caregiver burden/strain and HRQoL will be the primary outcome measures.

#### Secondary outcome

Caregivers’ knowledge of cerebral palsy and psychosocial indices such as depression, anxiety, stress, and self-efficacy will be the secondary outcome measures.

### Risk of bias (or “quality”) individual studies

Although random control trials (RCTs) are considered as the gold standard of evidence of efficacy of health care interventions, a preliminary search revealed a dearth of these study designs. We will consequently consider all quantitative designs, i.e., randomized controlled trials, quasi-experimental designs, case series, and comparable cohort/case control studies. To ensure internal and external validity of the selected studies, we will utilize The Briggs Institute checklists (see Additional files 1, 2, and 3) to assess the methodological quality of the studies. Two independent reviewers will assess the selected papers (CT and MTC) prior inclusion, and any disagreements will be resolved through discussions with a third independent reviewer (JJ).

### Best evidence synthesis

A preliminary/pilot search revealed a heterogeneity of caregivers’ well-being outcome measures. We will therefore perform a narrative/descriptive synthesis of the identified studies, as a meta-analysis would not be feasible. Where there is missing data, we will contact the researchers through email so that studies can be evaluated on the best available evidence.

## Discussion

Given the impact of provision of care for a child with a long-term physical disability/long-term health condition on the caregivers HRQoL, it is important to identify and evaluate the effects of interventions for improving caregivers’ well-being. This is essential as there is now a greater call for the improvement of the HRQoL of caregivers in addition to improving the HRQoL and functional outcomes in children with disabilities. This review will thus inform health care practitioners and researchers alike of the most practical and feasible methods of improving caregivers’ well-being within the confines of available resources. Further, findings can be used to design holistic and multimodal interventions aimed at improving caregivers’ well-being.

## Abbreviations

CINAHL, Cumulative Index of Nursing and Allied Health Literature; CP, cerebral palsy; HRQoL, health-related quality of life; PRISMA-P, Preferred Reporting Items of Systematic Reviews and Meta-Analyses Protocol; RCT, randomized controlled trials; UCT, University of Cape Town

## Additional files

Additional file 1: JBI critical appraisal checklist for randomised control/pseudo-randomised trial. (DOC 113 kb) Additional file 2: JBI critical appraisal checklist for descriptive/case series. (DOC 107 kb) Additional file 3: JBI critical appraisal checklist for comparable cohort/case control. (DOC 107 kb)
