# Supplementary material for: A critical evaluation of the effectiveness of interventions for improving the well-being of caregivers of children with cerebral palsy: a systematic review protocol
Source: Syst Rev. 2016 Jul 13;5:112. doi: 10.1186/s13643-016-0287-4 (PMC4944422; doi:10.1186/s13643-016-0287-4)

**Additional file 1: JBI Critical Appraisal Checklist for Randomised Control/ Pseudo-randomised Trial**


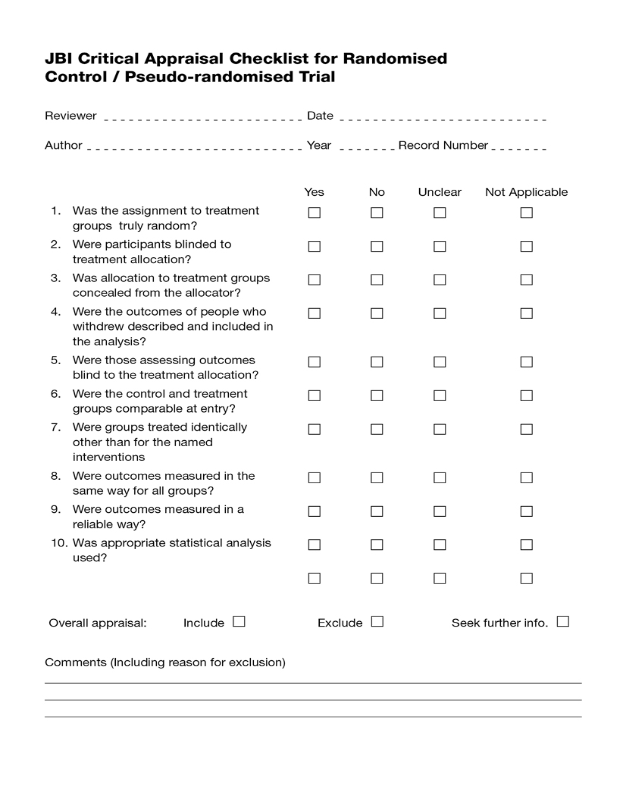

Supplement: Additional file 1: — JBI critical appraisal checklist for randomised control/pseudo-randomised trial. (DOC 113 kb) [file 13643_2016_287_MOESM1_ESM.doc]
