# Supplementary figures and images for: A critical evaluation of the effectiveness of interventions for improving the well-being of caregivers of children with cerebral palsy: a systematic review protocol
Source: Syst Rev. 2016 Jul 13;5:112. doi: 10.1186/s13643-016-0287-4 (PMC4944422; doi:10.1186/s13643-016-0287-4)

**Additional file 2: JBI Critical Appraisal Checklist for Descriptive/ Case Series**
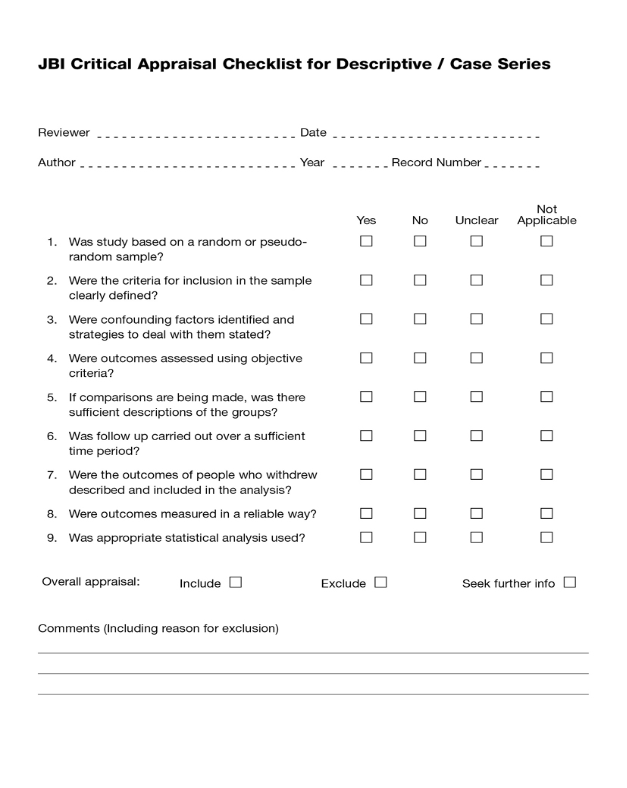

Supplement: Additional file 2: — JBI critical appraisal checklist for descriptive/case series. (DOC 107 kb) [file 13643_2016_287_MOESM2_ESM.doc]

**Additional file 3: JBI Critical Appraisal Checklist for comparable Cohort/ Case Control**


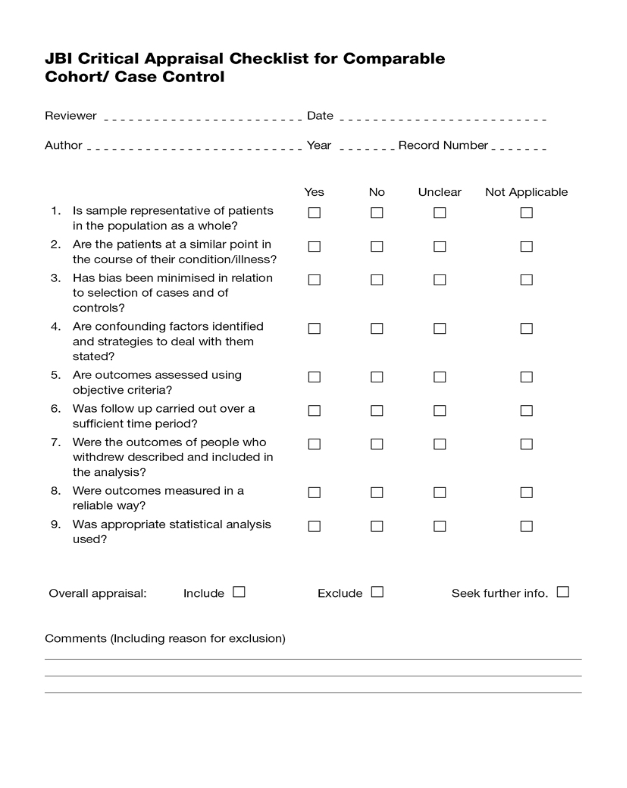

Supplement: Additional file 3: — JBI critical appraisal checklist for comparable cohort/case control. (DOC 107 kb) [file 13643_2016_287_MOESM3_ESM.doc]
